# p-hydroxy benzaldehyde attenuates intestinal epithelial barrier dysfunction caused by colitis via activating the HNF-1β/SLC26A3 pathway

**DOI:** 10.3389/fphar.2024.1448863

**Published:** 2024-11-22

**Authors:** Meng Liu, Yuhui Wang, Xiaotian Xu, Guoqiang Guan, Shu Zhang, Shengnan Zhu, Yang Liu, Yizhun Zhu, Xiaoqun Duan

**Affiliations:** ^1^ School of Pharmacy, Faculty of Medicine, Macau University of Science and Technology, Macau SAR, China; ^2^ School of Pharmacy, Guilin Medical University, Guilin, China; ^3^ School of Biomedical Industry, Guilin Medical University, Guilin, China

**Keywords:** ulcerative colitis, p-hydroxy benzaldehyde, intestinal epithelial barrier dysfunction, HNF-1β, SLC26A3

## Abstract

**Background:**

Intestinal epithelial barrier dysfunction is intricately linked to the pathogenesis of ulcerative colitis (UC). Dietary interventions that bolster intestinal epithelial barrier function can effectively thwart UC onset. Our prior research revealed that p-Hydroxy benzaldehyde (HD), a phenolic compound from Nostoc commune (an edible cyanobacterium), markedly upregulated the expression of E-cadherin, a pivotal protein in intestinal mucosa, thereby mitigating mucosal damage in mice afflicted with dextran sulfate sodium (DSS)-induced colitis. Nevertheless, the precise molecular mechanisms underpinning HD’s ameliorative effects on intestinal epithelial barrier dysfunction remain elusive.

**Methods:**

Dextran sodium sulfate (DSS)-induced colitis mouse model was established, and the successful establishment of the model was determined by evaluating the changes in body weight, disease activity index (DAI), colonic histopathology, and white blood cell count. Transmission electron microscopy (TEM) observed the ultrastructural changes of intestinal villi. The levels of inflammatory factors ( IFN-γ IL-13 ) and intestinal permeability indicators (FITC-Dextran, DAO, ET, and D-LA ) were detected by Enzyme-linked immunosorbent assay (ELISA). Western blotting (WB) and immunohistochemistry (IHC) were used to detect the expression of intestinal barrier integrity-related factors such as tight junction protein TJs (ZO-1, occludin) and adhesion junction protein AJs (E-cadherin). Furthermore, WB, Pull-down assay, drug affinity reaction target stability (DARTS) assay, molecular docking and molecular dynamics (MD) simulation were used to determine the potential target and molecular mechanism of HD.

**Results:**

HD intervention significantly alleviated the symptoms of colitis mice, inhibited the weight loss and colon shortening, reduced DAI score and colon pathological score, maintained the ultrastructure of intestinal villi in colon tissue, and significantly reduced the inflammatory factors IFN-γ, IL-13 and the number of white blood cells in colon tissue of colitis mice. HD could also reduce the levels of FITC-Dextran, DAO, ET, and D-LA and increase the expression of ZO-1, occludin, and E-cadherin in the colonic tissues of colitis mice, thereby maintaining the impaired intestinal barrier function caused by colitis. Mechanically, HD augmented the expression of hepatocyte nuclear factor 1β (HNF-1β) and DRA. Adeno-associated virus (AAV)-HNF-1β shRNA or Lentivirus-mediated HNF-1β knockdown effectively abolished HD-induced intestinal barrier protection, as well as the promotion of solute carrier family 26 member 3 (SLC26A3) expression levels. SLC26A3 siRNA effectively reversed the inhibition of intestinal permeability by HD. Pull-down assay, DARTS analysis, molecular docking, and MD results showed high binding strength, interaction efficiency and remarkable stability between HNF-1β and HD.

**Conclusion:**

This study elucidates HD’s role in forestalling intestinal epithelial barrier disruption under colitis conditions. Mechanistic investigations revealed that HD fortifies TJs and AJs expression via the HNF-1β/SLC26A3 pathway, thus preserving the lower intestinal epithelial barrier’s integrity in UC.

## 1 Introduction

Ulcerative colitis (UC) is an idiopathic gastrointestinal disease characterized by diarrhea and bloody stools (Rahm, 2024). Since the advent of the 21st century, alterations in dietary habits, particularly the adoption of a Western diet characterized by high sugar and fat content coupled with low fiber, have been associated with an increasing prevalence of UC (Ahadi et al., 2024). Although the widespread use of biological drugs in the past decade has increased the clinical remission rate of UC, the long-term efficacy has not increased significantly, and biological drugs are expensive, which has brought a heavy economic burden to public health (Long, 2024). Therefore, it is imperative to find safe and effective candidate drugs that can be used for the prevention and treatment of UC.

In recent years, several natural products have been recognized for their unique multi-target actions, good tolerability and low-cost attracting much attention (Zhou et al., 2023). Natural products are abundant in nature and generate numerous structurally diverse secondary metabolites, including a large number of small molecule compounds with anti-inflammatory activity (Zhou et al., 2023). *Nostoc commune*, a cyanobacterium vegetable with significant nutritional benefits, is recognized for its anticancer, anti-inflammatory, and antimicrobial attributes (Li and Guo, 2018). Our previous research has shown that p-hydroxy benzaldehyde (HD), a phenolic compound from *N. commune*, can relieve colitis, reduce inflammation, and increase E-cadherin expression (Xu et al., 2021). Yet, the exact molecular mechanisms through which HD ameliorates intestinal epithelial barrier dysfunction remain to be elucidated. Studies have shown that E-cadherin is closely related to mucosal barrier function, mucin 2 (MUC2) and goblet cell number. E-cadherin loss interferes with goblet cell maturation and migration (Schneider et al., 2010), reducing the number of goblet cells, thereby reducing the secretion of mucin MUC2 and damaging mucosal barrier function (Liu M. et al., 2023). Based on these findings, this study aims to elucidate HD’s protective effects on the intestinal epithelial barrier and its mechanisms by developing *in vitro* and *in vivo* models of intestinal barrier injury, thereby assessing its therapeutic potential for UC.

## 2 Materials and methods

### 2.1 Drugs and reagents

DSS (molecular weight: 36–50 kDa) was acquired from MP Biomedicals (Solon, United States). HD (C_7_H_6_O_2_, molecular weight: 122.12, purity, ≥98%) was sourced from Yuanye Biology (Shanghai, China). Recombinant tumor necrosis factor-alpha (TNF-α) was supplied by R&D Systems, Inc. (California, United States). IFN-γ (120062), and interleukin 13 (IL-13) enzyme-linked immunosorbent assay (ELISA) kits were purchased from Dakowei Biotech Co., Ltd. (Beijing, China). The concentrations of serum diamine oxidase (DAO, CSB-E10090m) and endotoxin (ET, CSB-E13066m) ELISA kits were from Cusabio LLC. (Houston, TX, United States). The D-lactate (JL48176) ELISA Kit was obtained from Shanghai Future Industrial Co., Ltd., Shanghai, China. Rabbit monoclonal antibodies against targeting E-cadherin (#AF0131), ZO-1 (#AF5145), occludin (#DF7504), and HNF-1β (#AF5394) were provided by Affinity Biosciences (Minnesota, United States). The antibody for DRA (ab83545) were sourced from Abcam (Shanghai, China). All chemicals utilized in this study were of analytical or chromatographic grade.

### 2.2 Induction of colitis in murine model and drug administration

Eight-week-old male specific pathogen-free C57BL/6 mice, weighing 18–22 g, were obtained from Hunan Selleck Jingda (license number: SCXK Xiang 2021-0002). The mice were maintained in an environment with a constant temperature (20°C–26°C), humidity (45%–65%), and a 12-h light/dark cycle. The study design and methods received approval from the Animal Ethics Committee of Guilin Medical University (approval number GLMC202103003). Acute colitis was induced by administering 2.5% DSS in drinking water for 7 days, followed by 3 days of normal drinking water. On the 11th day, all mice were euthanized.

To assess the impact of HD on colitis, mice underwent a week of acclimatization and were then randomly divided into four groups: normal, DSS, and DSS + HD (at doses of 3, 6, and 12 mg/kg, respectively; n = 6 per group). Mice in the treatment groups received the designated drugs via gavage once daily for 10 days. Mice in the normal and DSS-only groups received deionized water orally via gavage at a volume of 0.1 mL/10 g.

To investigate HNF-1β′s role in HD’s alleviation of colitis-induced epithelial barrier dysfunction, HNF-1β gene knockdown and control Adeno-associated virus-green fluorescence protein vectors 9 (AAV9-GFP) were procured from Genechem (Shanghai Genechem Co., Ltd.). The mice were allocated into six groups (n = 6 each): normal group; DSS group; DSS + adeno-associated virus (AAV)-scramble shRNA group; DSS + AAV-HNF-1β shRNA group; DSS + HD (12 mg/kg) +AAV-scramble shRNA group; DSS + HD (12 mg/kg) +AAV-HNF-1β shRNA group. A week prior to the experiment, 200 μL of AAV-scramble shRNA (5 × 10^10^) and AAV-HNF-1β shRNA (5 × 10^10^) viral particles in PBS were administered via tail vein injection into C57BL/6 mice. HD was dissolved in PBS and delivered orally through gavage daily throughout the study. The normal and DSS groups were administered an equivalent volume of the vehicle for 10 consecutive days. At the study’s conclusion, mice were euthanized, and their colons were harvested for analysis.

### 2.3 Assessment of disease activity index (DAI) scores, colon length, myeloperoxidase (MPO) activity, white blood cell (WBC) counts and pathological alterations

Observations and recordings of body weight, diarrhea, and hematochezia were conducted daily to determine DAI scores. The scoring criteria in Supplementary Table S1. It was calculated as the average of these three parameters. At the experiment’s conclusion, the mice were euthanized, and analyses of colon length, MPO activity, peripheral blood WBC counts, and pathological alterations were performed.

### 2.4 Assessment of intestinal barrier integrity *in vivo*

#### 2.4.1 Examination of intestinal villi ultrastructure via transmission electron microscopy (TEM)

Post-euthanasia, colon tissues were promptly immersed in 2.5% glutaraldehyde and stored at 4°C. Subsequently, tissues were fixed in 2.5% glutaraldehyde for 2 h, washed thrice in 0.1 mmol/L phosphate buffer, then fixed in 1% osmium tetroxide for 2 h, followed by another three washes in phosphate buffer. After dehydration, infiltration, and embedding, sections were stained and examined by transmission electron microscopy (H-7650, Hitachi, Japan).

#### 2.4.2 Assessment of intestinal permeability using FITC-dextran, DAO, ET, and D-Lactate

The procedure from our previous study was employed (Liu Y. et al., 2023). Briefly, after a 4-h fast, 0.3 mL of FITC-Dextran (6 mg/10 g body weight) was administered orally to each mouse. Blood samples were collected 3 h later, centrifuged (3,000 r/min, 4°C, 10 min) to obtain serum. Serum fluorescence intensity was measured in a black 96-well plate (excitation at 485 nm and emission at 535 nm).

Subsequent measurements of DAO, ET, and D-lactate levels were conducted using ELISA kits according to the manufacturer’s instructions.

### 2.5 Cell culture and treatment

The Caco-2 cell line, acquired from the Kunming Cell Bank, which was cultured in high-glucose Dulbecco’s modified Eagle medium (DMEM) (Solarbio, Beijing, China) supplemented with 10% fetal bovine serum (FBS) and 1% penicillin-streptomycin, maintained at 37°C in 5% CO_2_. To maintain cell growth consistency, experiments were conducted between passages 10 and 25. Post-adherence, cells underwent a 4-h starvation period followed by co-treatment with HD and TNF-α (10 ng/mL) for 24 h.

HNF-1β and scramble shRNA were procured from Shanghai Genome Biotech. Lentiviral transfection into Caco-2 cells was performed as per the provided guidelines, using polybrene (10 μg/mL) as a transduction adjuvant. The culture medium was replaced after 24 h, and transfection continued for an additional 48 h before cell collection.

siDRA and siControl were obtained from RiboBio (Guangzhou, China), with the siRNA targeting sequence for DRA being GCU​GGA​CAA​CAA​UCA​GAU​ATT. Caco-2 cells were plated in 6-well plates at 40%–60% confluency 24 h before transfection. Transient transfection was carried out using Lipofectamine 2000 (Invitrogen; Thermo Fisher Scientific, Inc.), following the manufacturer’s protocol.

### 2.6 Evaluation of Caco-2 mucosal barrier injury

#### 2.6.1 Measurement of transepithelial electrical resistance (TEER)

TEER is indicative of the ionic current resistance associated with cell monolayer integrity, inversely correlating with cellular integrity. Caco-2 cells (1 × 10^5^ cells/well, 0.4 mL) were seeded in Transwell chambers with a 0.4 µm pore size and cultured until they formed monolayers. These were then subjected to TNF-α (10 ng/mL) or a specified HD dose for 24 h. Measurements were conducted using a Millicell ERS-2 voltmeter (Millipore). Post-electrode equilibration, the shorter end was placed in the upper chamber and the longer end in the lower chamber. By stabilizing the electrode, readings from three random points in each chamber were obtained and calculated using the formula: TEER = (Measured resistance− Control resistance) (Ω) × 0.33 cm^2^.

#### 2.6.2 FITC-dextran permeability assay

After reaching monolayer formation in Transwell chambers (TEER of Caco-2 cells reached >400 Ω cm^2^), the cells were cultured in serum-free medium for 12 h. Next, the monolayer was treated with vehicle (control), TNF-α (10 ng/mL), or TNF-α with HD (0, 1, 3, or 10 μM) for 24 h. Subsequently, monolayers were rinsed three times with Hank’s balanced salt solution (HBSS), and then 100 μg/mL FITC–dextran was added, while the lower medium was replaced by HBSS. Following a 1-h incubation at 37°C, 200 μL from the lower chamber was sampled, and fluorescence intensity was assessed using a multimodal microplate reader. Fluorescein levels were ascertained by referencing a standard curve.

### 2.7 Phalloidin staining

The cytoskeleton was visualized through F-actin staining using YF^®^ 594-phalloidin YF^®^594 (YP0052S/YP0052L, Chemstan, Wuhan, China). In brief, cells were fixed with 4% paraformaldehyde in PBS for 20 min at room temperature, followed by permeabilization with 0.4% Triton X-100 in PBS for 10 min. After three washes with PBS, cells were stained with diluted YF^®^ 594-phalloidin in PBS for 20 min and then with DAPI for 5 min at room temperature. Following two PBS washes, images were captured under a microscope for analysis.

### 2.8 Western blot (WB) analysis

T Proteins from colonic tissues or Caco-2 cells were extracted using RIPA lysis buffer (Beyotime Biotechnology Co., Beijing, China), separated by SDS-PAGE, and transferred to nitrocellulose (NC) membranes. These membranes were blocked with 5% skim milk and incubated overnight at 4°C with primary antibodies against ZO-1, occludin, E-cadherin, HNF-1β, and DRA. After incubation with appropriate secondary antibodies for 2 h at room temperature, bands were visualized using a ChemiDoc™ XRS + imaging system (Bio-Rad Laboratories, Inc., California, United States). Protein band intensity was quantified using ImageJ software (Bio-Rad Laboratories, Inc., California, United States).

### 2.9 Immunofluorescence (IF)

Caco-2 cells (2 × 10^5^ cells/well) were seeded in 24-well plates and incubated for 12 h, followed by co-treatment with HD (1, 3, 10 μM) and TNF-α (10 ng/mL) for 24 h. The cells were fixed with 4% paraformaldehyde for 30 min, permeabilized with 0.2% Triton X-100 for 20 min, and blocked with 5% bovine serum albumin (BSA) for 30 min at room temperature. After overnight incubation at 4°C with primary antibodies against ZO-1 and E-cadherin (1 : 200), cells were treated with Alexa Fluor^®^ 488 conjugated anti-rabbit IgG for 1.5 h at room temperature the next day, followed by staining with 4,6-diamidino-2-phenylindole (DAPI, 5 μg/mL) for 10 min. Imaging was performed using an Olympus microscope (Tokyo, Japan).

### 2.10 Pull-down assay

As described in the previous report (Wang et al., 2022), intestinal homogenates were prepared using ice-cold RIPA buffer with 1% protease inhibitors. Protein solutions were then incubated with HD-bound beads or control beads in the presence or absence of HD (1, 3, 10 μM). The beads were washed five times with PBS containing 0.01% Triton X-100. Proteins captured on the beads were pulled down and detected by Western blot using specific antibodies.

### 2.11 Drug affinity responsive target stability (DARTS) assay

As previously reported, the interaction between HD and HNF-1β was examined using the DARTS assay (Lomenick et al., 2009). Briefly, Caco-2 cells, either treated or untreated with HD (10 μM) for 1 h, were lysed using 600 μL of M-PER mammalian protein extraction reagent (Cat#78503, Pierce, Thermo Fisher Scientific, Inc.) on ice. The lysate supernatant was obtained by centrifugation at 10,000 rpm for 10 min, the lysate supernatant was split into two aliquots and each was incubated with pronase for 30 min. Subsequently, HNF-1β protein expression was assessed by WB.

### 2.12 Statistical analysis

Data were analyzed with GraphPad Prism 9.0 software. Results from various experimental groups were expressed as mean ± standard error of the mean (SEM). Normality tests were performed to verify the distribution of data, followed by one-way analysis of variance (ANOVA) and Dunnett’s multiple comparisons test for statistical analyses. Statistical significance was set at *P* < 0.05.

## 3 Results

### 3.1 HD alleviates DSS-induced colitis in mice

**FIGURE 1:**
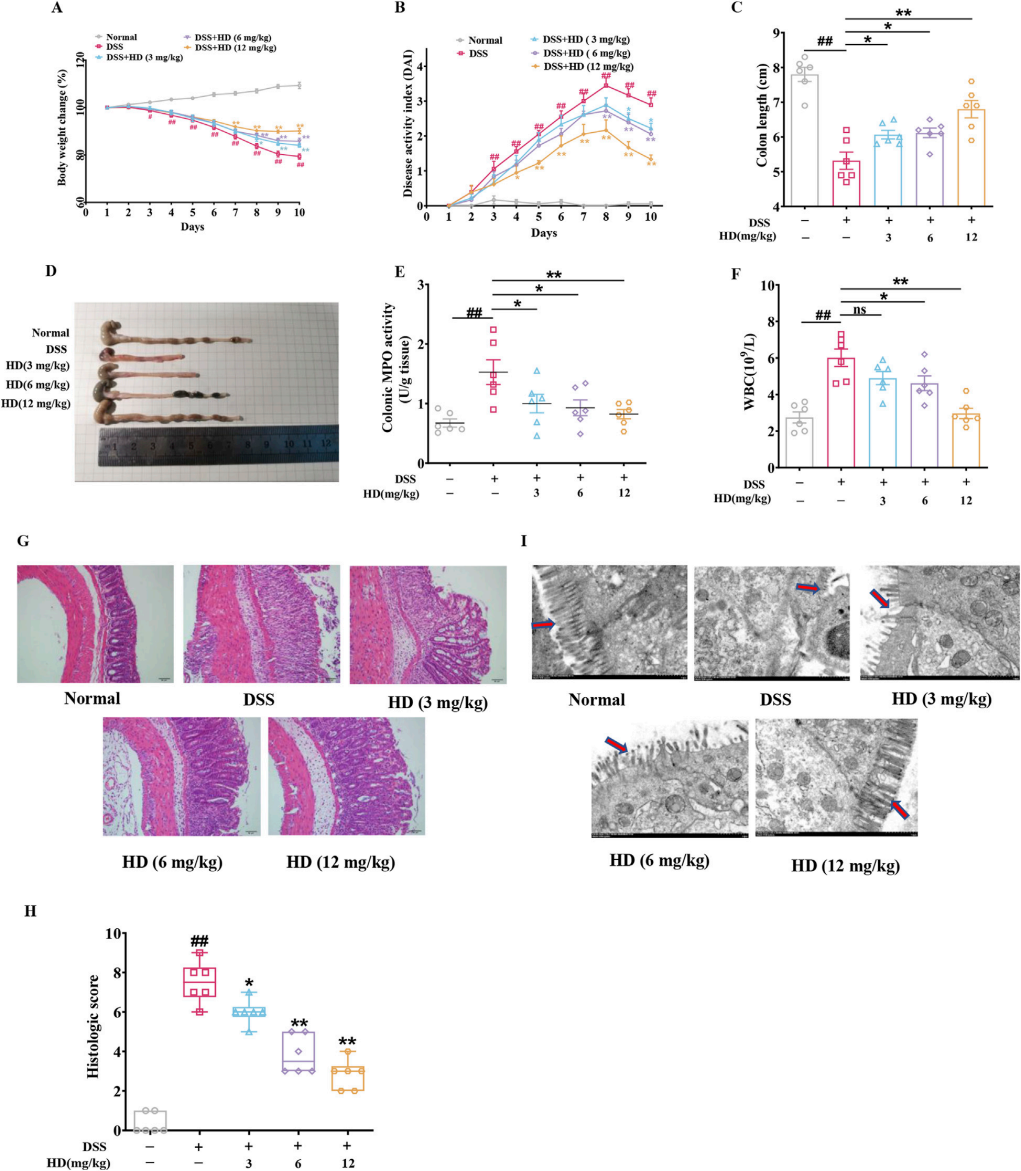
Impact of HD on symptom amelioration in DSS-induced acute colitis in mice. Mice with enteritis received oral HD administration (3, 6, and 12 mg/kg) daily for 10 days **(A)** Changes in body weight percentage among the groups. **(B)** DAI scores for the respective groups. **(C)** Measurements of colon length. **(D)** Illustrative colon images. **(E)** Colon MPO activity was assessed according to the manufacturer’s protocol. **(F)** Effects of HD on WBC counts in colitis-afflicted mice. **(G)** Exemplary H&E-stained colon tissue sections. Scale bar, 50 μm. **(H)** Histopathological evaluation. **(I)** Characteristic ultrastructural images of mouse intestinal villi. Scale bar, 500 μm. Results are expressed as mean ± SEM (n = 6). ^#^ *P* < 0.05, ^##^ *P* < 0.01 vs. the normal group; ^*^ *P* < 0.05. ^**^ *P* < 0.01 vs. the DSS group, and ns indicates *P* > 0.05.

HD (3, 6, and 12 mg/kg) was administered orally to mice with colitis via gavage over a 10-day period. Relative to the control group, the DSS-treated mice exhibited progressive weight loss, diarrhea, and hemorrhage, culminating in a marked elevation in DAI score and reduced colon length. HD administration (3, 6, and 12 mg/kg) resulted in a dose-dependent augmentation of body weight (Figure 1A, *P* < 0.05), decrease in DAI score (Figure 1B, *P* < 0.05), and extension of colon length (Figure 1C, *P* < 0.05). MPO, indicative of neutrophil infiltration, was notably decreased in HD-treated groups (6 and 12 mg/kg) (Figure 1E), along with a reduction in peripheral blood WBC counts in colitis mice (Figure 1F), signifying mitigated inflammation (*P* < 0.05, vs. model). Moreover, H&E staining revealed organized and intact colonic mucosa, villi, and crypts in the control group, whereas these structures were disrupted in the DSS group. Notably, HD treatment (3, 6, and 12 mg/kg) significantly ameliorated tissue damage in the colon, as evidenced by markedly lower histopathological scores compared to DSS-treated mice (*P* < 0.05, Figure 1H). Histological scoring was performed independently by two pathologists who were blinded to treatment history as described previously (Charlet et al., 2020). Additionally, TEM analysis demonstrated that, unlike the disordered villi in the DSS group, villus architecture was relatively preserved post-HD treatment (Figure 1I). These findings indicate that HD counteracts DSS-induced intestinal inflammation in mice.

### 3.2 HD reduced intestinal permeability in mice with DSS-induced enteritis

**FIGURE 2:**
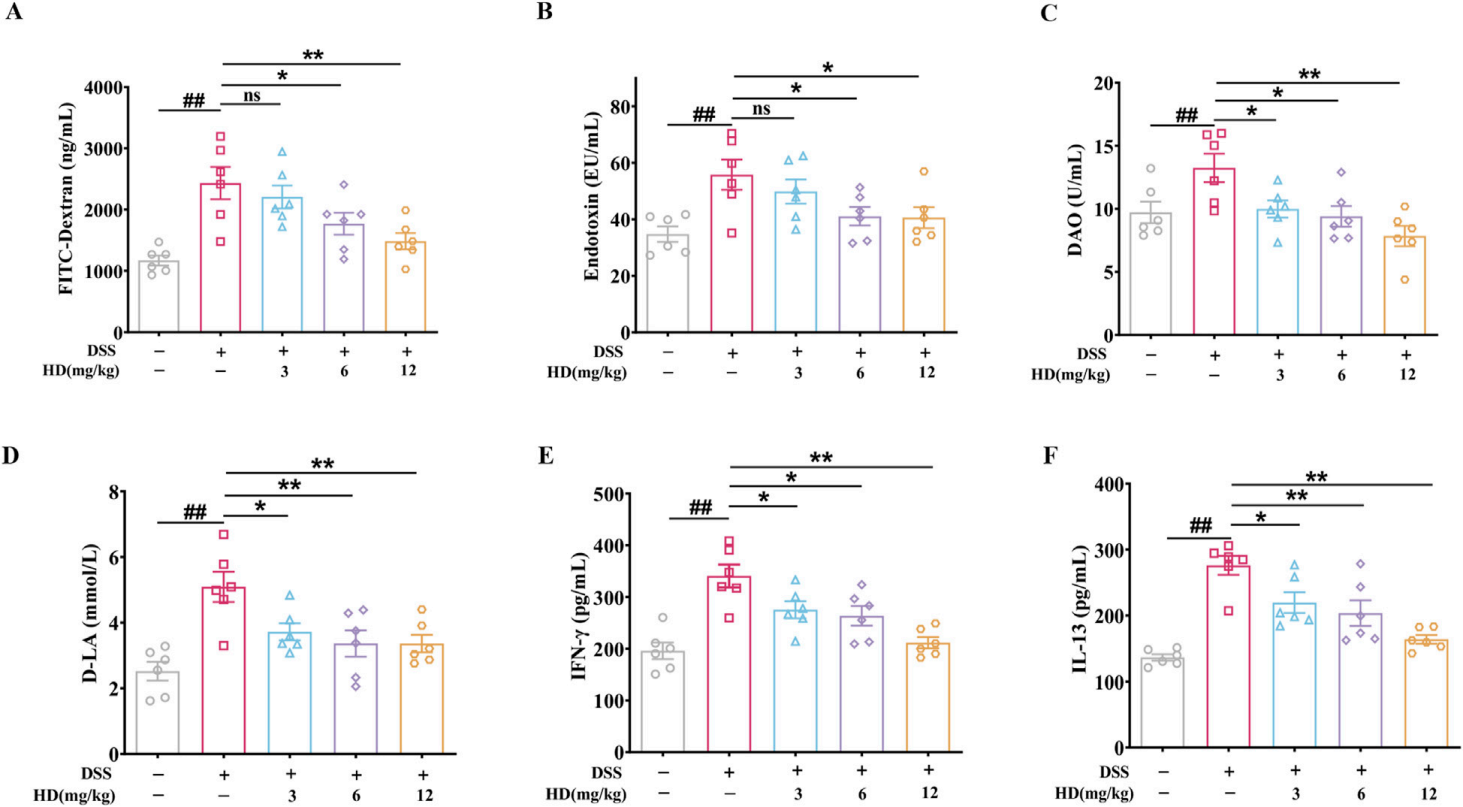
HD attenuates intestinal permeability in mice with DSS-induced enteritis. Mice afflicted with enteritis received daily oral doses of HD (3, 6, and 12 mg/kg) for 102 days **(A)** FITC-Dextran levels. **(B)** ET concentrations. **(C)** DAO levels. **(D)** D-LA concentrations. **(E)** IFN-γ and **(F)** IL-13 levels in colonic homogenates were assessed using ELISA. Data are expressed as mean ± SEM (n = 6). ^#^ *P* < 0.05, ^##^ *P* < 0.01 vs. the normal group; *P < 0.05. **P < 0.01 vs. the DSS group, and ns indicates *P* > 0.05.

Intestinal barrier dysfunction and elevated intestinal permeability are key characteristics of UC, with increased serum levels of FITC-glucan, DAO, D-LA, and ET typically indicating barrier impairment. Our findings reveal that DSS administration escalated serum FITC-Dextran, ET, DAO, and D-LA levels by 1.08 -, 0.6 -, 0.38 - and 1.02 - fold, respectively (*P* < 0.01, vs. normal), indicating significant intestinal barrier disruption and enhanced permeability in the enteritis model. Conversely, mice with colitis treated with HD (12 mg/kg) showed reduced damage to the intestinal barrier, as evidenced by decreases in FITC-Dextran (Figure 2A), ET (Figure 2B), DAO (Figure 2C), and D-LA (Figure 2D) serum levels by 51.9%, 27.3%, 41.5%, and 33.9%, respectively. This reduction signifies that HD supplementation curtails the leakage of ET, DAO, and D-LA into the bloodstream, thus safeguarding intestinal mucosal integrity. Notably, HD at 3 mg/kg significantly curbed DAO and D-LA but not ET levels, highlighting a dose-dependent effect of HD.

Furthermore, the association between increased IL-13 and IFN-γ levels and TJ disruption, contributing to barrier impairment in colitis, is well-documented (Huang et al., 2020). ELISA results indicated that HD markedly reduced IFN-γ (275.40 ± 16.42, 263.60 ± 18.85, 211.80 ± 10.86 in the HD group vs. 340.80 ± 22.15 in the model group; Figure 2E) and IL-13 (219.50 ± 15.74, 203.80 ± 19.4, 163.90 ± 6.60 in the HD group vs. 276.1 ± 14.37 in the model group; Figure 2F) levels. These outcomes provide preliminary evidence that HD attenuates intestinal epithelial permeability, underscoring its potential in bolstering barrier protective functions.

### 3.3 HD enhances the integrity of tight junction proteins (TJs) and adhesion junctions (AJs) in the intestinal epithelium of mice with colitis

**FIGURE 3:**
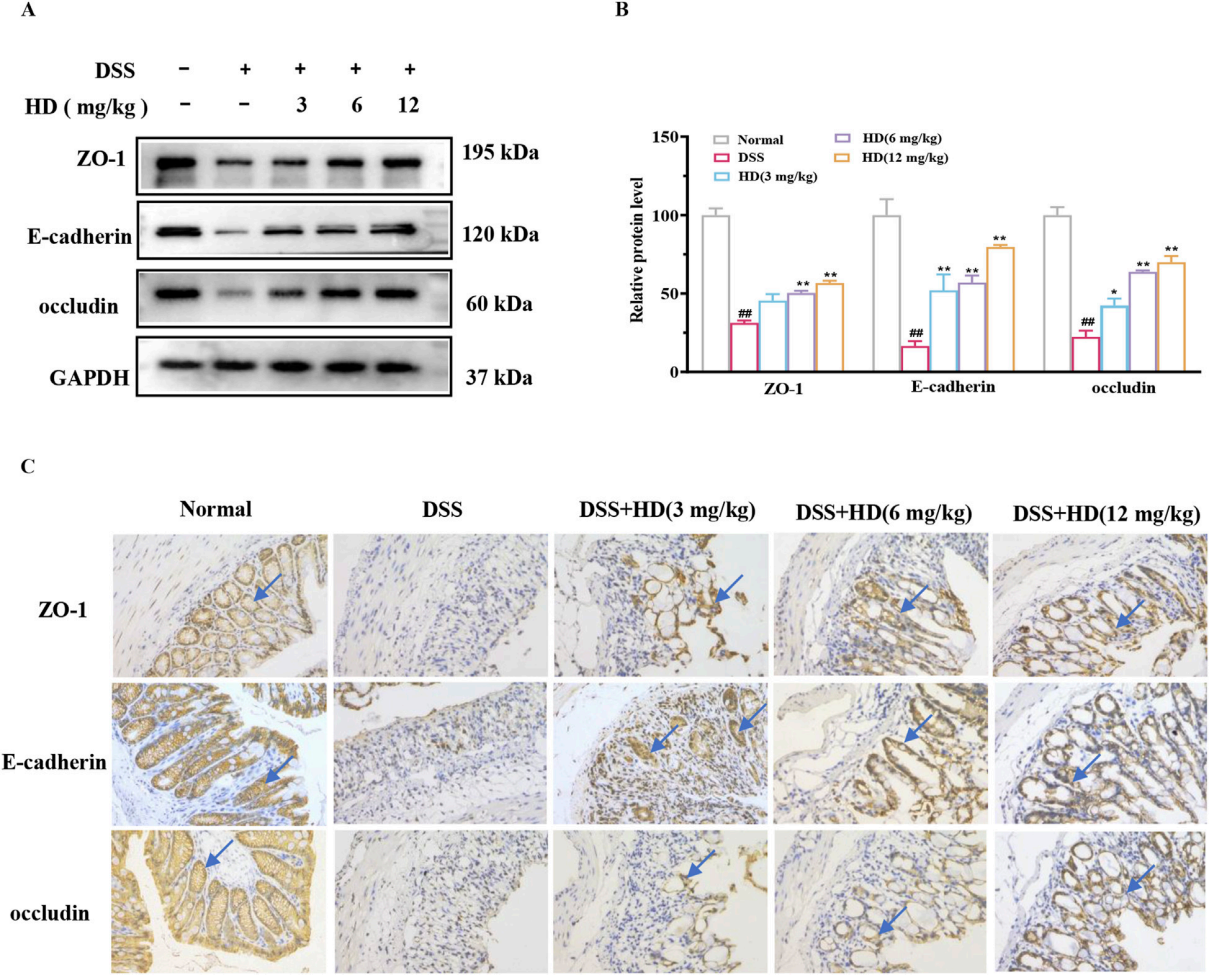
HD augments the expression of TJs and AJs in mice with DSS-induced acute colitis. Mice afflicted with enteritis received oral HD (3, 6, and 12 mg/kg) daily for 10 days **(A)** Western blot analysis of ZO-1, occludin, E-cadherin, and GAPDH in colon tissues. GAPDH as a control. **(B)** Densitometric evaluation of ZO-1, occludin, and E-cadherin. **(C)** Immunohistochemistry assessed the expression of ZO-1, occludin, and E-cadherin. Scale bar, 20 μm. Results are expressed as mean ± SEM (n = 3). ^#^ *P* < 0.05, ^##^ *P* < 0.01 vs. the normal group; *P < 0.05. **P < 0.01 vs. the DSS group, and ns indicates *P* > 0.05.

The integrity of the apical junctional complex (AJC), comprising TJs (such as ZO-1 and occludin) and AJs (such as E-cadherin), is crucial for intestinal permeability (Laukoetter et al., 2008). We assessed the impact of HD on these essential proteins in the intestinal epithelium of mice with enteritis. As depicted in Figure 3, the expression levels of ZO-1, occludin, and E-cadherin were significantly decreased in the colon tissues of the DSS group mice compared to the normal group (*P* < 0.01). However, treatment with HD (6, 12 mg/kg) markedly enhanced the expression of ZO-1, occludin, and E-cadherin relative to the DSS group (*P* < 0.05, Figures 3A, B). Immunohistochemistry corroborated the Western blot results, indicating diminished expression of ZO-1, E-cadherin, and occludin in the DSS group, which was reversed following HD treatment (Figure 3C). These findings imply that HD may bolster intestinal barrier function by upregulating TJs and AJs.

### 3.4 HD mitigates TNF-α-induced barrier dysfunction *in vitro*

**FIGURE 4:**
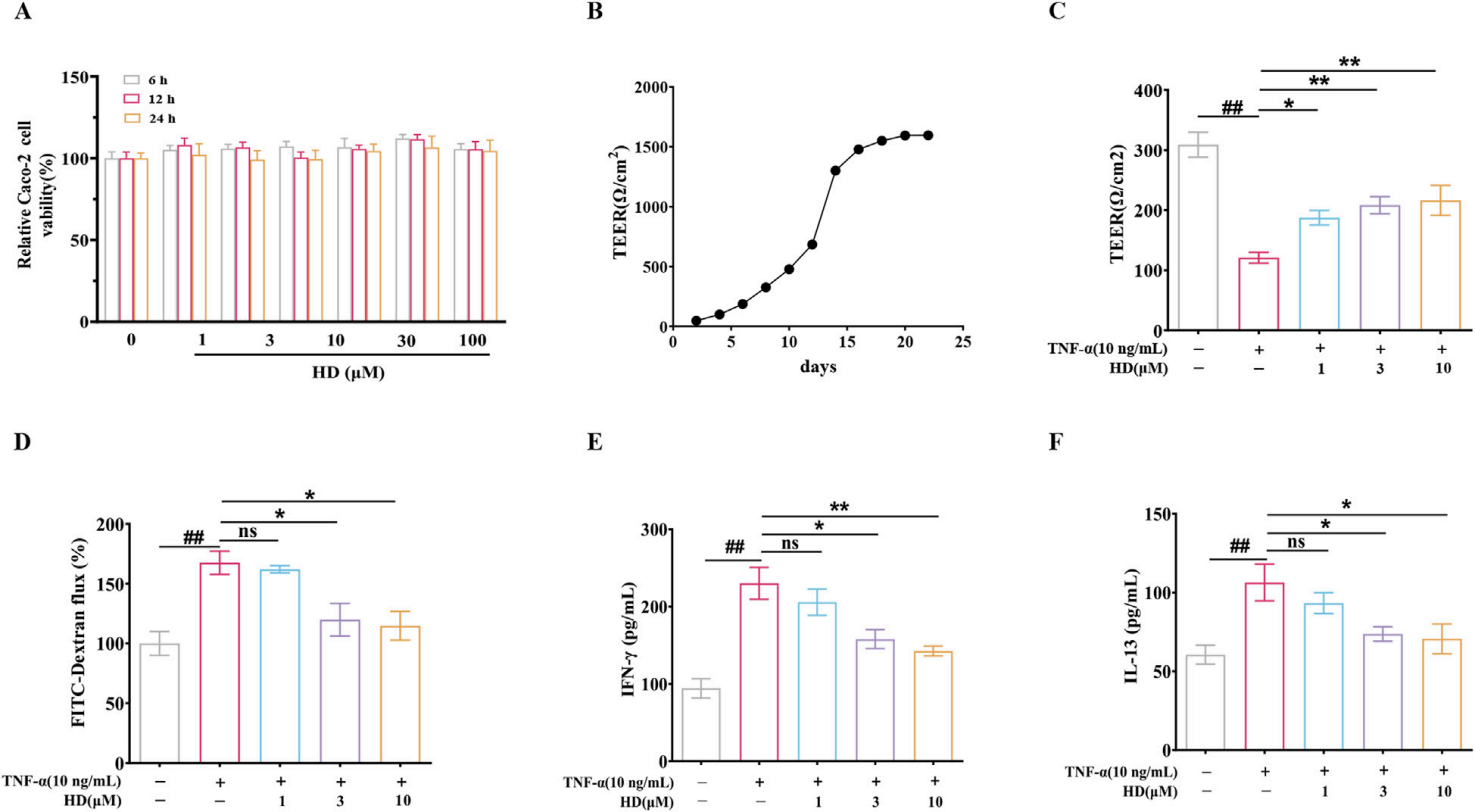
**(A)** Caco-2 cell viability was assessed using the CCK-8 assay (n = 6). **(B)** Caco-2 cells were cultured in Transwell chambers for 21 days to form a monolayer of colonic epithelial cells. **(C, D)** TEER and FITC-Dextran flux measurements were conducted. **(E, F)** Cytokine levels (IFN-γ/IL-13) in the supernatants of Caco-2 cell cultures were quantified by ELISA (n = 4). Data are presented as mean ± SEM, from 3 independent experiments. ^##^ *P* < 0.01 vs. the control group and ^*^ *P* < 0.05, ^**^ *P* < 0.01 vs. the TNF-α (10 ng/mL) group, and ns indicates *P* > 0.05.

To ascertain if HD’s protective effect is attributable to a direct influence on epithelial cells, we investigated HD’s impact on epithelial barrier function *in vitro* using TNF-α-stimulated Caco-2 monolayer cells. Cytotoxicity assays revealed that Caco-2 cell viability was altered after exposure to various HD concentrations (0–100 μM) over different durations (6, 12, and 24 h), yet no significant deviation from the control was observed (*P* > 0.05, Figure 4A). For experimental efficiency and cost-effectiveness, we selected HD concentrations of 1, 3, and 10 μM for a duration of 24 h for further analysis.

Subsequently, intestinal epithelial barrier integrity was assessed by measuring TEER and FITC-Dextran flux. Caco-2 cells were cultured in Transwell chambers for 21 days to form a colonic epithelial monolayer (Figure 4B). HD notably counteracted the TNF-α-induced decrease in TEER (187.50 ± 11.91, 208.30 ± 14.06, 216.30 ± 24.96 vs. 120.90 ± 9.08; Figure 4C). Additionally, we evaluated HD’s effect on the paracellular tracer FITC-Dextran permeability in TNF-α-exposed Caco-2 monolayers. Aligning with TEER results, TNF-α augmented FITC-Dextran cellular passage by 0.68 – fold (*P* < 0.01, vs. control), which HD effectively inhibited (3.3%, 28.5%, 31.5% reduction). Moreover, HD (3 and 10 μM) diminished the expression of two cytokines, TNF-α and IL-13, associated with intestinal permeability (*P* < 0.05, Figures 4E, F). These findings indicate that HD directly safeguards the epithelial barrier functionality of Caco-2 cell monolayers against TNF-α-induced disruption.

### 3.5 HD supplementation enhanced TNF-α-induced expression of TJs and AJs proteins and improved the localization of cytoskeletal F-actin

**FIGURE 5:**
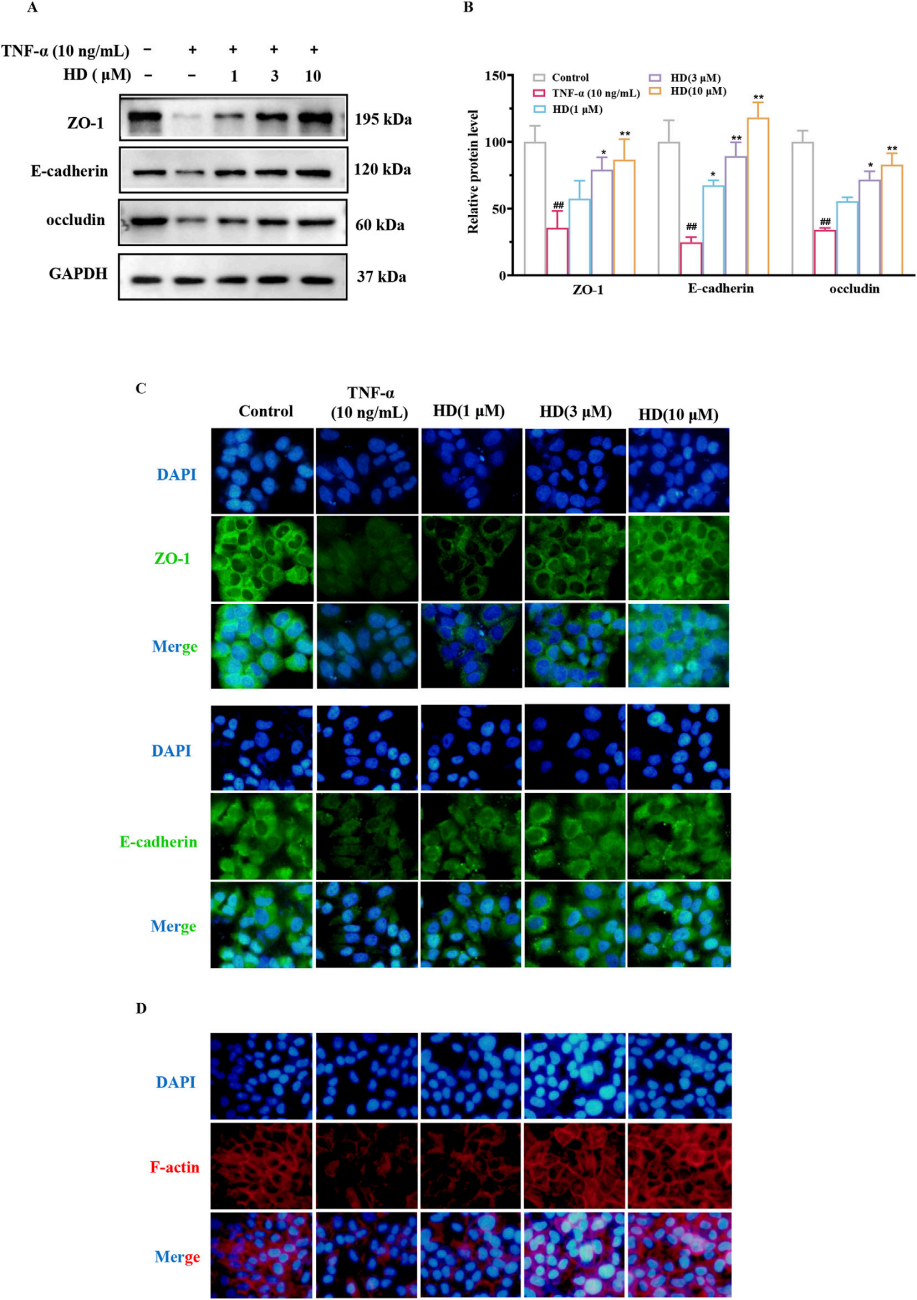
Effect of HD on TJs, AJs, and F-actin expression in Caco-2 cells by WB and IF. Caco-2 cells were treated with TNF-α (10 ng/mL), and TNF-α (10 ng/mL) + HD (1, 3, and 10 μM) for 24 h. **(A)** Shows representative WB images of ZO-1, occludin, E-cadherin, and GAPDH in Caco-2 cells. **(B)** Presents densitometric analysis of ZO-1, occludin, and E-cadherin. **(C)** Illustrates ZO-1 and E-cadherin levels based on IF staining (×200 magnification). Scale bar, 50 μm. **(D)** Depicts Caco-2 cell monolayers stained for F-actin using phalloidin (×200 magnification). Scale bar, 50 μm. Results are expressed as mean ± SEM, from 3 independent experiments. ^##^ *P* < 0.01 compared with the control group, ^*^ *P* < 0.05, and ^**^ *P* < 0.01 compared with the TNF-α (10 ng/mL) group, while ns indicates *P* > 0.05.

The protein expression levels of ZO-1 (*P* < 0.01), occludin (*P* < 0.01), and E-cadherin (*P* < 0.01) in Caco-2 cells were significantly reduced after TNF-α stimulation (vs. control) compared to the control group, but recovery was observed following HD supplementation (3 and 10 μM), suggesting (vs. TNF-α) that HD mitigates the TNF-α-induced damage to TJs and AJs barriers (Figures 5A, B), particularly at higher HD concentrations. Furthermore, the correct localization of TJs and AJs proteins is essential for barrier integrity. Previous research indicates TNF-α disrupts the localization of TJs and AJs, compromising the barrier function of Caco-2 cells (Huang et al., 2020). Our study confirms that TNF-α alters the distribution of ZO-1 and E-cadherin in Caco-2 cells, whereas HD treatment (1, 3, 10 μM) prevents these abnormalities (Figure 5C). Additionally, TNF-α was found to cause F-actin disarray in the Caco-2 cell monolayer. Our findings reveal that TNF-α-induced F-actin disassembly was inhibited by HD (Figure 5D). These results collectively indicate that HD counters TNF-α-induced alterations in localization patterns.

### 3.6 HD activated the HNF-1β/DRA pathway

**FIGURE 6:**
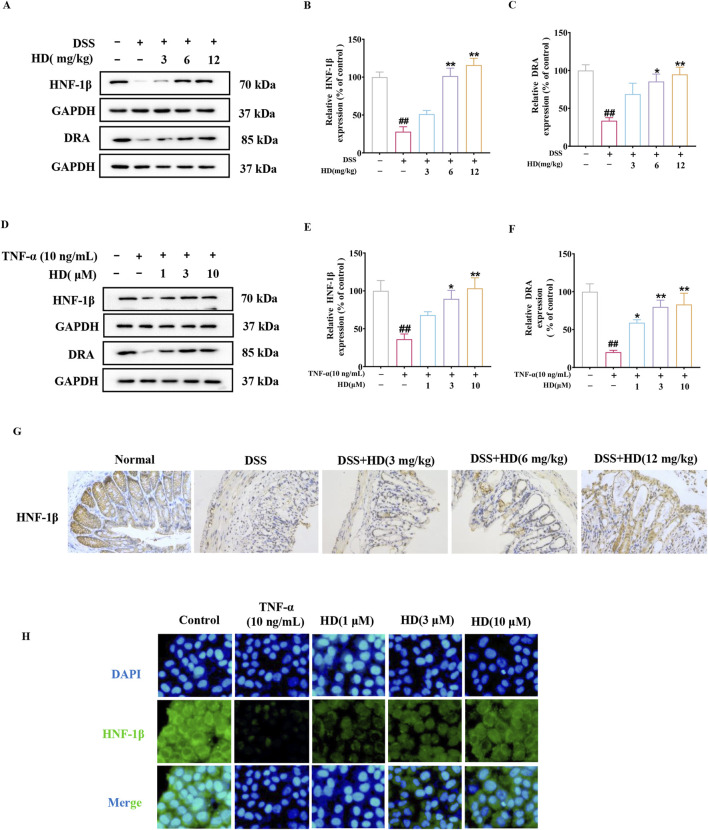
HD activated the HNF-1β/DRA pathway. Mice with enteritis received oral HD (3, 6, and 12 mg/kg) daily for 10 days. Caco-2 cells underwent treatment with TNF-α (10 ng/mL), and TNF-α (10 ng/mL) + HD (1, 3, and 10 μM) for 24 h **(A, D)** Show representative WB of HNF-1β, DRA, and GAPDH in colon tissues and Caco-2 cells (n = 3). **(B, C, E, F)** Provide densitometric analysis of HNF-1β and DRA proteins. **(G)** Immunohistochemistry assessed HNF-1β expression. Scale bar, 20 μm. **(H)** HNF-1β levels via IF staining (×200 magnification, n = 3). Scale bar, 50 μm. Results are presented as mean ± SEM from 3 independent experiments. ^##^ *P* < 0.01 compared to the normal or control group, ^*^ *P* < 0.05, ^**^ *P* < 0.01 compared to the DSS or TNF-α (10 ng/mL) group, with ns indicating *p* > 0.05.

The chloride transporter SLC26A3, known as the adenoma downregulated protein (DRA), plays a crucial role in intestinal anion exchange, facilitating luminal fluid absorption and maintaining acid/base balance (Yu, 2021). DRA is integral to the protection of the intestinal epithelial barrier and mitigates inflammation processes associated with TJs and AJs (Ding et al., 2018). Previous research has demonstrated that Hepatocyte nuclear factor 1β (HNF-1β) can bind to the promoter region of the DRA gene, thereby regulating its transcription. Considering these findings and HNF-1β role in modulating TJ and AJ-related proteins (Ferrè and Igarashi, 2019), we proposed that HD could protect the intestinal barrier by activating the HNF-1β/DRA pathway. Western blot analysis revealed that HD treatment (6 and 12 mg/kg) significantly elevated HNF-1β protein expression levels (Figures 6A, B) compared with the model group, a result corroborated by immunohistochemistry (Figure 6G). Furthermore, HD notably increased the expression of DRA, a downstream factor of HNF-1β, particularly at the higher HD dose (12 mg/kg) (*P* < 0.01).

Consistent with *in vivo* studies, our hypothesis was corroborated by an *in vitro* model of TNF-α-induced intestinal barrier damage. Figures 6D, E shows that HD treatment significantly elevated the protein expression levels of HNF-1β (3 and 10 μM, *P* < 0.05), a finding reinforced by immunofluorescence observations (Figure 6H). Moreover, DRA levels increased in a dose-responsive manner upon HD administration, especially at the higher dosage (10 μM, Figures 6D, F), indicating the involvement of the HNF-1β/DRA pathway in intestinal protection by HD.

### 3.7 DRA knockdown abolished the intestinal protective effects of HD

**FIGURE 7:**
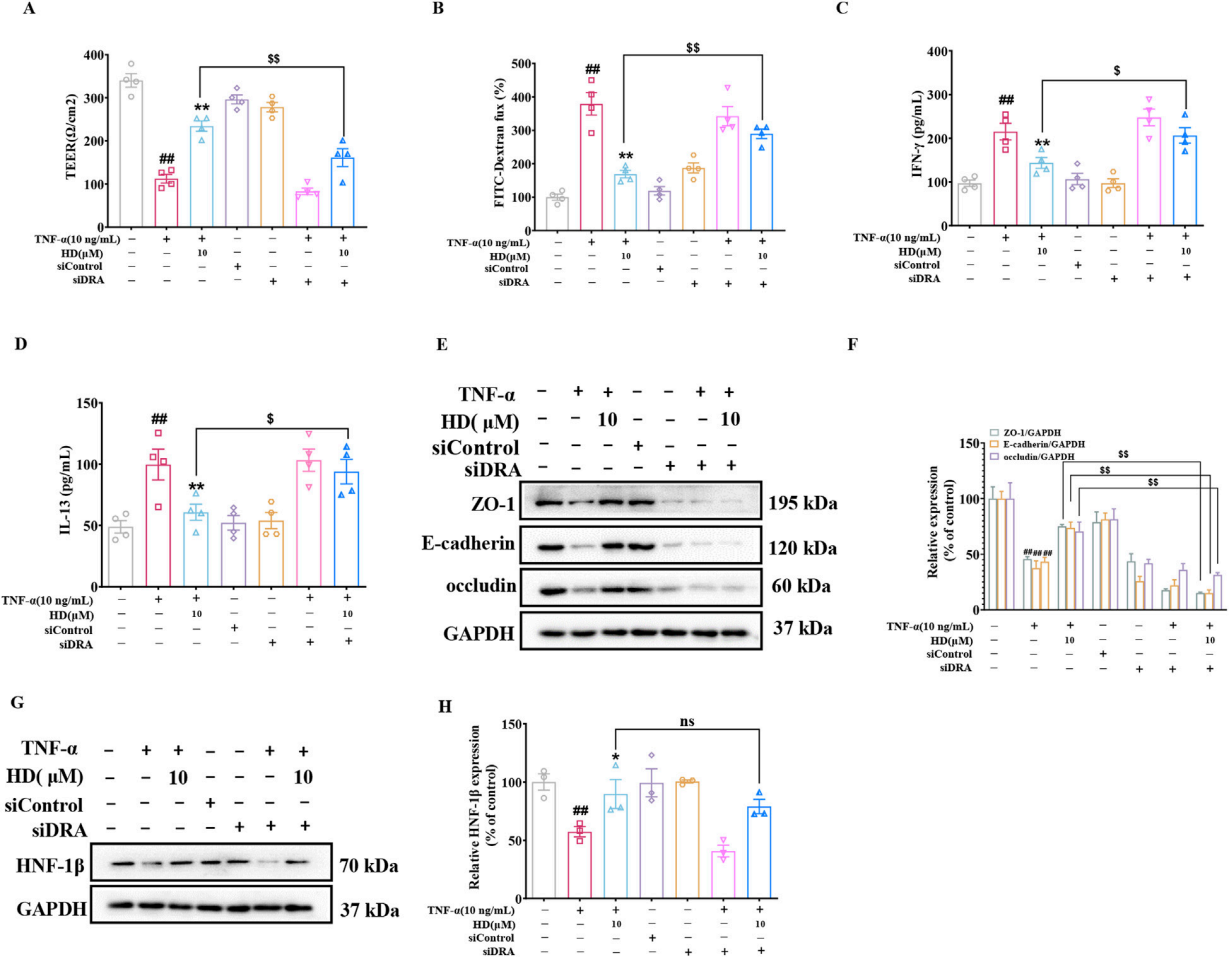
DRA’s role in HD’s intestinal barrier protection. Post-DRA siRNA transfection, Caco-2 cells were treated with TNF-α (10 ng/mL) and TNF-α (10 ng/mL) + HD (10 μM) for 24 h **(A)** TEER and **(B)** FITC-dextran flux were measured, **(C, D)** Cytokine levels (IFN-γ/IL-13) in the supernatants via ELISA (n = 4). **(E)** WB images for ZO-1, E-cadherin, occludin, and GAPDH in Caco-2 cells. **(F)** Densitometric analysis of ZO-1, E-cadherin, and occludin. **(G)** Representative WB images of HNF-1β and GAPDH in Caco-2 cells. **(H)** Densitometric analysis of HNF-1β. Data, presented as mean ± SEM from 3-4 independent experiments. ^##^ *P* < 0.01 compared with the control group and ^*^ *P* < 0.05, ^**^ *P* < 0.01 compared with the TNF-α (10 ng/mL) group, ^$^ *P* < 0.05, ^$$^ *P* < 0.01 compared with the TNF-α (10 ng/mL) + HD (10 μM) group.

To explore the role of DRA in the protective influence of HD on intestinal epithelial barrier integrity, DRA was silenced in Caco-2 cells using siRNA interference. The findings revealed that HD (10 μM) counteracted the TNF-α-induced reduction in TEER and the associated increase in IL-13, IFN-γ, and FITC-Dextran permeability. However, DRA silencing markedly reduced HD’s efficacy in enhancing TEER (Figure 7A) and decreasing IFN-γ (Figure 7C), IL-13 (Figure 7D), and FITC-Dextran (Figure 7B) permeability (*P* < 0.05). Subsequently, we examined the impact of DRA silencing on the expression of TJs and AJs protein in Caco-2 cells using WB analysis. As illustrated in Figures 7E, F, siDRA-mediated DRA knockdown effectively counteracted the increase in ZO-1, occludin, and E-cadherin protein levels induced by HD treatment. Moreover, siRNA interference did not affect HNF-1β expression (*P* > 0.05, Figures 7G, H), suggesting DRA acts downstream of HNF-1β. Overall, DRA’s crucial role in HD-mediated protection of the intestinal epithelial barrier is highlighted by the attenuation of HD’s beneficial effects following DRA knockdown.

### 3.8 HNF-1β knockdown effectively reversed the intestinal protective effects of HD

**FIGURE 8:**
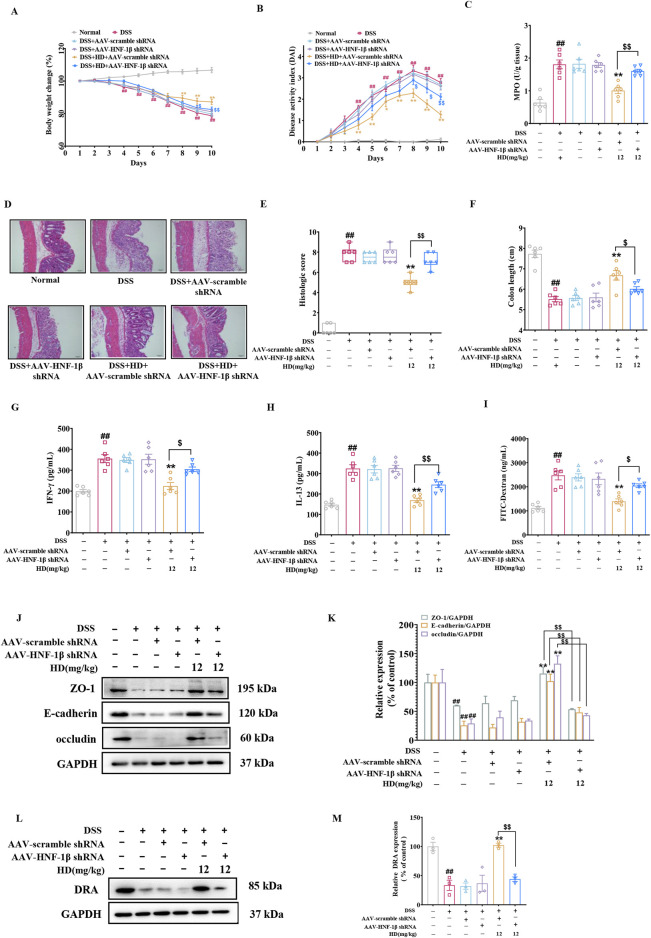
Underscores HNF-1β′s indispensable role in HD’s intestinal barrier protection. One week before the model induction, C57BL/6 mice received a tail vein injection of 200 μL of 5 × 10^10^ AAV physical particles in PBS, followed by oral HD administration (12 mg/kg) daily for 10 days to mice with enteritis. **(A)** Weight loss was observed in each group of mice. **(B)** The DAI was recorded for each group. **(C)** MPO activity in colon tissues was assessed as per the instructions provided with the assay kit. **(D)** Representative H&E stained images of colon tissue. Scale bar, 50 μm. **(E)** Pathohistological scores. **(F)** The lengths of the colons were measured in each group. **(G)** Permeability was assessed using FITC-Dextran. **(H)** IFN-γ and **(I)** IL-13 levels in colonic homogenates were quantified using ELISA (n = 6). **(J)** Representative WB images of tight junction proteins ZO-1, E-cadherin, occludin, alongside GAPDH as a control, in colonic tissue. **(K)** Provides densitometric quantification of ZO-1, E-cadherin, and occludin levels. **(L)** Shows representative WB images of DRA and GAPDH in colonic tissues. **(M)** Details densitometric analysis of DRA expression. Data are presented as mean ± SEM from 3 independent studies. ^##^ *P* < 0.01 indicates significant difference from the normal group, while ^*^ *P* < 0.05, ^**^ *P* < 0.01 signify differences from the DSS-treated group. ^$^ *P* < 0.05 and ^$$^ *P* < 0.01 denote significant changes relative to the DSS + AAV-scramble shRNA + HD (12 mg/kg) group.

To assess HNF-1β role in restoring the intestinal barrier with HD treatment, we administered 200 μL of 5 × 10^10^ AAV physical particles in PBS via tail vein injection into C57BL/6 mice 1 week prior to the experiment to silence HNF-1β. It was observed that the primary beneficial effects of HD (12 mg/kg) on colitis improvement were offset by AAV-HNF-1β shRNA (*P* < 0.05, Figures 8A–F). The impact of HD on intestinal permeability-related inflammatory cytokines (IFN-γ and IL-13) was significantly reversed by AAV-HNF-1β shRNA (*P* < 0.05, Figures 8G, H). Similarly, HD’s reduction of FITC-Dextran permeability was reversed following HNF-1β knockdown (*P* < 0.05, Figure 8I). As anticipated, while HD (12 mg/kg) enhanced the expression of TJs (ZO-1, occludin) and AJs (E-cadherin), AAV-HNF-1β shRNA administration diminished these increases (*P* < 0.01, Figures 8J, K). Additionally, the silencing of HNF-1β significantly reduced the HD-induced upregulation of DRA expression (*P* < 0.01, Figures 8L, M).

**FIGURE 9:**
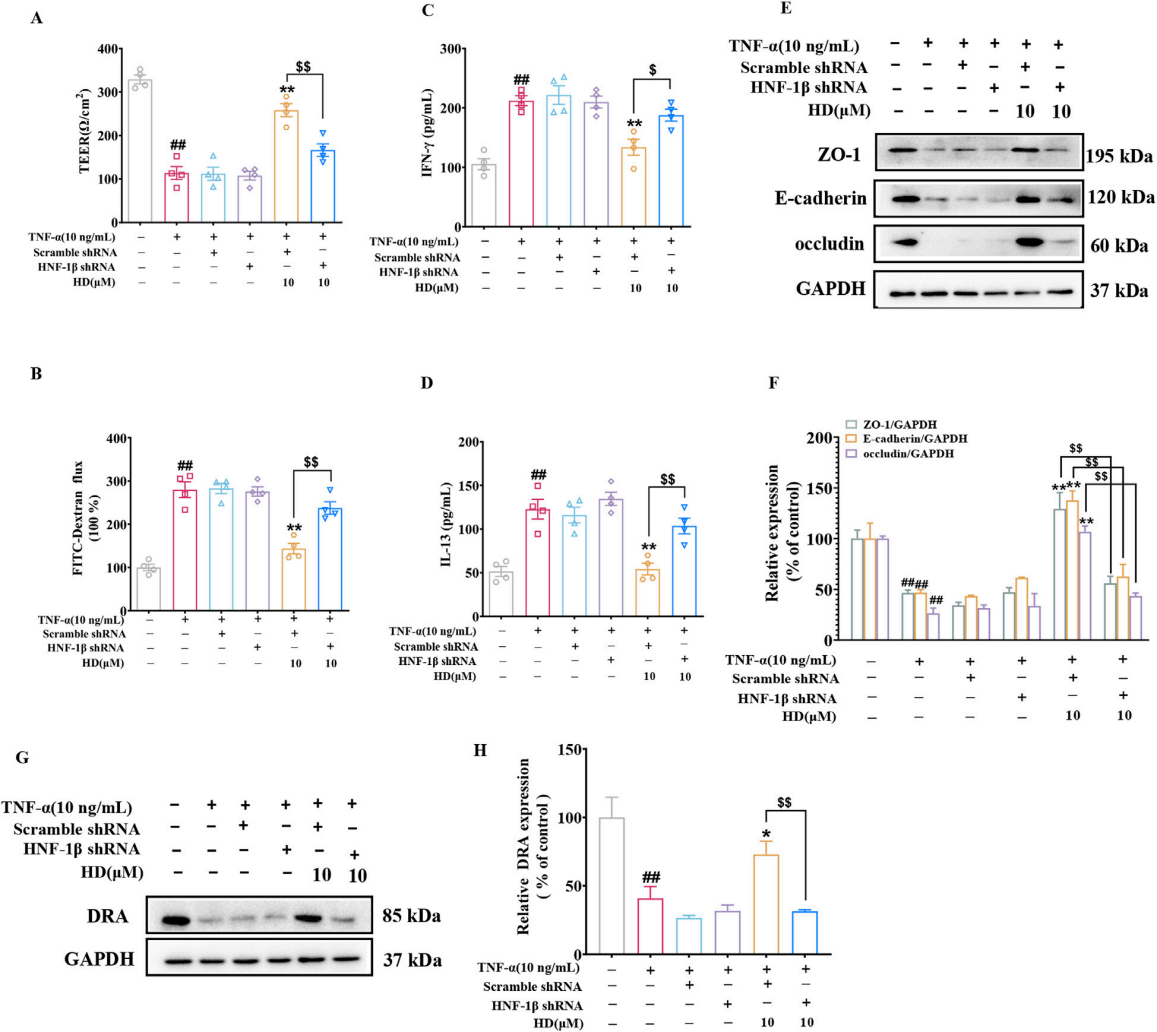
Illustrates HNF-1β′s significance in HD-induced protection of the intestinal barrier in Caco-2 cells. After transfection with lentiviral HNF-1β shRNA, Caco-2 cells were exposed to TNF-α (10 ng/mL) alone or combined with HD (10 μM) for 24 h **(A)** TEER and **(B)** FITC-Dextran flux were assessed, **(C, D)** along with cytokine levels (IFN-γ/IL-13) in cell culture supernatants, determined via ELISA (n = 4). **(E)** Displays WB images of tight junction proteins ZO-1, E-cadherin, occludin, and the control protein GAPDH in Caco-2 cells. **(F)** Presents densitometric quantification of ZO-1, E-cadherin, and occludin. **(G)** Shows WB images of the DRA protein and GAPDH in Caco-2 cells. **(H)** Provides densitometric analysis of DRA expression. Data are represented as mean ± SEM, based on 3-4 independent experiments. ^##^ *P* < 0.01 signifies a significant difference from the control group, ^*^ *P* < 0.01 and ^**^ *P* < 0.01 indicate significant differences compared to the TNF-α (10 ng/mL) group, and ^$^ *P* < 0.05, ^$$^ *P* < 0.01 denote significant differences when compared with the TNF-α (10 ng/mL) + HD (10 μM) group.

To validate the role of HNF-1β-mediated upregulation of DRA expression in enhancing HD’s protective effects on the epithelial barrier, we utilized a lentiviral assay encoding HNF-1β shRNA. Consistent with *in vivo* findings, silencing HNF-1β in Caco-2 cells markedly reduced the HD-induced enhancements in intestinal epithelial barrier function (Figures 9A–F) and the upregulation of DRA expression (Figures 9G, H). These results support the conclusion that HD improves DRA expression and rectifies intestinal epithelial barrier defects through an HNF-1β-dependent mechanism.

### 3.9 HD functioned as an HNF-1β agonist

**FIGURE 10:**
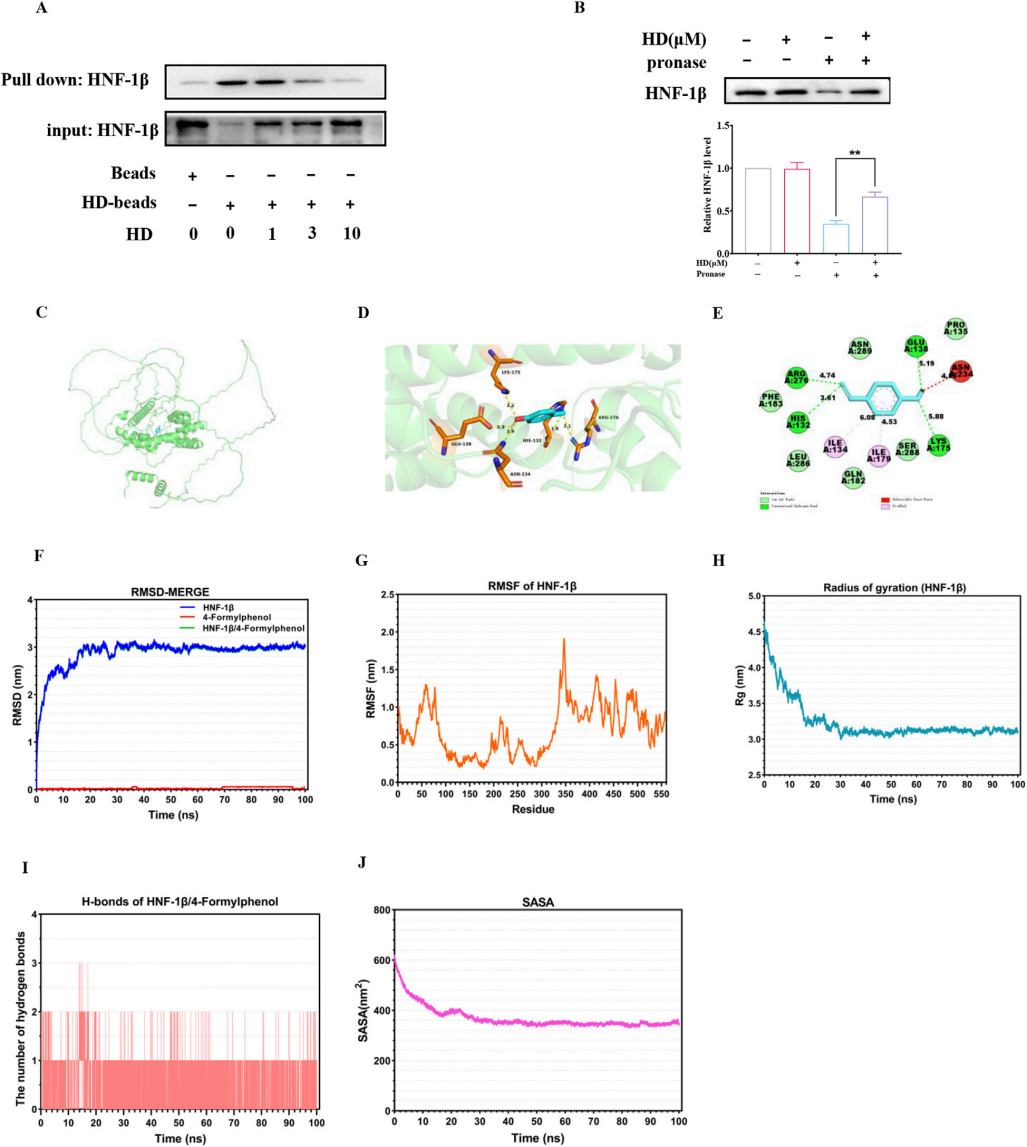
Illustrates the direct interaction between HD and HNF-1β. **(A)** Intestinal tissue lysates were incubated with HD-coated beads, followed by a pull-down assay. Immunoblots using a specific HNF-1β antibody are displayed (n = 3). **(B)** The DARTS assay assessed HD’s effect on HNF-1β′s susceptibility to proteolysis. **(C)** Shows the optimal docking model between HD and HNF-1β. **(D)** The HD-HNF-1β complex (3D plot) was visualized and analyzed with PyMol. **(E)** The HD-HNF-1β complex (2D plot) was visualized and analyzed using Discovery Studio visualizer software. **(F)** RMSD plots illustrate the stability of HD (red line), HNF-1β (blue line), and the HNF-1β-HD complex (green line). **(G)** RMSF plots detail the flexibility of residues within HNF-1β. **(H)** Rg plots for HNF-1β indicate the compactness of the protein structure. **(I)** Displays the number of hydrogen bonds within the HD-HNF-1β complex. **(J)** SASA plots for the HD-HNF-1β complex highlight the surface area accessible to solvents. Results are expressed as mean ± SEM from 3 independent experiments. ^**^ *P* < 0.01, indicating statistical significance when compared with the pronase-treated group.

Our exploration into whether HD’s activation of the HNF-1β/DRA signaling pathway was contingent upon its interaction with HNF-1β involved conducting pull-down assays to verify the physical connection between HD and HNF-1β. The assays demonstrated that HNF-1β was successfully pulled down by HD-coated beads in intestinal tissue lysates, revealing that HD binding inversely correlated with concentration (Figure 10A). Moreover, DARTS experiments indicated that proteolytic degradation of HNF-1β by proteases was significantly reduced in the presence of HD (Figure 10B), hinting at HD’s potential to enhance HNF-1β′s resistance to proteolysis through direct interaction.

In the realm of computer-assisted drug design, molecular docking studies are instrumental in predicting binding efficiencies and interaction dynamics between proteins and ligands. Our study employed the CB-Dock two platform for a molecular docking analysis to gauge the binding affinity between HD and HNF-1β. The optimal docking configuration, showcasing the interaction between HD and HNF-1β, was illustrated in Figure 10C, with a binding affinity calculated as −5.3 kcal/mol. Further visualization and analysis of the HD-HNF-1β complex through PyMol (3D plot) and Discovery Studio visualizer (2D plot) revealed that HD established a hydrogen bond with HNF-1β at residues GLU138, ASN234, HIS132, ARG276, and LYS175. Additionally, HD maintained stable van der Waals interactions with residues PHE183, LEU286, GLN182, SER288, and PRO135, and engaged in pi-alkyl interactions with residues ILE134 and ILE179 for robust binding to HNF-1β (Figure 10E).

Additionally, a 100 ns MD simulation was conducted to explore the dynamic properties of the HD-HNF-1β complex. The stability of chemical compounds and protein complexes is primarily assessed through MD simulation results, which include root mean square deviation (RMSD), root mean square fluctuation (RMSF), radius of gyration (Rg) value, solvent accessible surface area (SASA), and hydrogen bonds (H-bonds) analysis.

The mobility of the receptor-ligand pair was assessed using RMSD, which indicated structural changes in the protein. As shown in Figure 10F, the RMSD curve for the HD-HNF-1β complex stabilized around 3 nm from 30 to 100 ns. RMSF evaluated the variations within the complex at the residue level, revealing that residues 30-70, 200-230, 340-350, and 400-500 of HNF-1β exhibited increased flexibility (Figure 10G). The Rg value, indicative of protein volume and tertiary structure, offers insights into protein stability within biological systems. A higher Rg value suggests increased ligand flexibility and decreased system stability, whereas a lower Rg value indicates a compact and tightly packed structure. Figure 9H illustrates that the Rg value for the HNF-1β complex remained consistent during the 30–100 ns simulation, with the lowest mean Rg value ranging between 3.0 and 4.5 nm. Hydrogen bonding, a significant non-covalent interaction, showed that the HD-HNF-1β complex systems with stable hydrogen bonds (1-2) existed consistently throughout the MD simulation, with a maximum of 3 hydrogen bonds noted (Figure 10I). These bonds between the ligand and receptor enhance the complex’s stabilization. Furthermore, the SASA value for the HNF-1β protein remained steady throughout the simulation, indicating structural stability and minimal substantial structural alterations (Figure 10J). Collectively, these MD simulation outcomes suggest the HD-HNF-1β complex’s relative stability.

## 4 Discussion

Pharmacological therapy is currently the mainstay management for UC. However, food, as a daily consumed resource, offers a sustainable, and comprehensive alternative, providing patients with a more natural, integrated, and practical approach to enhancing intestinal health. Through suitable nutritional supplementation and dietary modifications, functional foods have the potential to protect and repair the intestinal mucosa, strengthen intestinal barrier function, and reduce the incidence of UC (Liu Y. et al., 2023). Food influences the UC process predominantly by modulating the function of the intestinal mucosal barrier. Excessive intake of high-glucose foods and animal protein can reduce intestinal mucus secretion and increase mucosal permeability, thus inflicting damage on the intestinal mucosa and precipitating UC (Tian et al., 2024). Furthermore, the overconsumption of beef, lamb, and similar foods can aggravate gastrointestinal stagnation, leading to recurrent symptoms such as abdominal pain, diarrhea, and the occurrence of bloody stool in UC patients (Wark et al., 2020). Numerous food additives found in processed foods, including carbonated beverages, can induce oxidative stress in intestinal epithelial cells, elevate epithelial permeability, and compromise the integrity of the intestinal epithelial barrier (Jani, 2023; Wu et al., 2021). In contrast, consuming foods high in dietary fiber, such as fruits and vegetables, can be advantageous for UC management. These foods can improve intestinal blood circulation, stimulate the secretion of gastrointestinal hormones, encourage the growth of colonic mucosal epithelial cells, aid in repairing intestinal mucosal damage, and decrease the frequency of UC episodes (Ao et al., 2024). Thus, preventing UC by ameliorating intestinal mucosal injuries, especially through the consumption of foods with restorative impacts on intestinal mucosal damage is attractive.

Our prior research indicated that HD, a food additive derived from *N. commune*, markedly elevated the expression of E-cadherin, a critical protein in the intestinal mucosa, thus mitigating intestinal mucosal injury in mice afflicted with DSS-induced colitis (Xu et al., 2021). Nonetheless, the molecular mechanisms through which HD ameliorates intestinal epithelial barrier dysfunction has so far been unclear. Building on our previous findings, this study corroborates the beneficial impact of HD from *N. commune* in counteracting DSS-induced damage to the intestinal barrier. Further investigation suggests that HD may rectify intestinal barrier dysfunction through HNF-1β/DRA-mediated enhancement of TJs and AJs proteins, thereby offering relief from UC. HD presents as a promising option for the prevention and treatment of UC, harboring the potential to change the current approach to UC management.

Intestinal epithelial cells are the primary constituents of the barrier. Under normal conditions, the apical junctional complex (AJC) preserves the structural integrity of the intestinal barrier and facilitates the paracellular transport of ions and molecules, thus maintaining elevated transepithelial electrical resistance (Li et al., 2022). The AJC comprises TJ (e.g., ZO-1, occludin) and AJ (e.g., E-cadherin) (Citi et al., 2024). Alterations in the distribution and abundance of TJ and AJ proteins can result in enhanced permeability of the intestinal epithelium and a disturbance in immune homeostasis. Yet, an intact AJC structure can effectively close intercellular gaps, blocking the intrusion of harmful substances into the submucosal area (Lee et al., 2023). Clinical studies have indicated that the expression levels of TJ and AJ proteins are reduced in the colonic mucosa of UC patients (El-Haggar et al., 2024). The decrease in TJ and AJ proteins in the intestine leads to increased intestinal permeability and contributes to the development and progression of UC, manifesting symptoms such as diarrhea and rectal bleeding. Consequently, safeguarding the AJC is vital for the restoration of the intestinal barrier and is crucial in UC treatment. A growing body of research underscores the significant protective role of dietary plants and phytochemicals on the intestinal mucosa. Portulaca oleracea L. (purslane) improved the intestinal barrier by regulating TJ proteins levels (Liu et al., 2024). (−)-Syringaresinol treatment increased the LPS-decreased levels of TJ proteins, reinstated TEER, and maintained the intestinal barrier’s integrity (Ning et al., 2024). Similar to previous studies, our current study shows that HD supplementation in both *in vitro* and *in vivo* models of DSS- and TNF-α-induced injury results in the recovery of decreased expression of TJ proteins (ZO-1 and occludin) and AJ protein (E-cadherin). Additionally, *in vitro* experiments revealed that HD reinstated TEER, diminished FITC-Dextran flux, and enhanced intestinal epithelial permeability in a TNF-α-induced Caco-2 monolayer injury model. In the pathogenesis of IBD, cytokines such as TNF-α, IFN-γ, and IL-13 can harm the intestinal epithelium, destroy the AJC, further compromising barrier integrity, and exacerbating inflammation (Cristina Lopes do Carmo et al., 2020), potentially playing a pivotal role in stubborn and chronic IBD cases. Here, HD decreased levels of IFN-γ and IL-13 in mice with colitis, contributing to its anti-inflammatory effect by improving intestinal epithelial barrier function. In summary, the evidence supports that HD effectively prevents intestinal epithelial dysfunction by modulating the expression of TJ and AJ proteins.

DRA plays a crucial role as a Cl^−^/HCO ^3−^exchanger in the human intestinal epithelium, vital for maintaining fluid and electrolyte absorption in the intestines (Lee and Hong, 2024). Dysfunction of DRA leads to congenital chloride diarrhea. Importantly, diminished DRA expression has been noted in animal models of colitis and in UC patients (Jayawardena et al., 2023). Additionally, genome-wide association studies have identified defects or mutations in the DRA gene linked to an elevated risk of UC (Asano et al., 2009). Beyond its known functions, recent studies have underscored DRA’s role in maintaining intestinal barrier homeostasis (Anbazhagan et al., 2024). DRA-KO mice and Caco-2 cells exhibit various intestinal anomalies, including increased paracolonic cell permeability and significantly lowered levels TJ (ZO-1, occludin) and AJs (E-cadherin). Conversely, delivery of DRA via an adenovirus vector markedly mitigated colitis progression and enhanced epithelial barrier function by fostering the recovery of TJ and F-actin proteins in DSS-treated mice (Ding et al., 2018). These findings underscore DRA’s positive impact on the intestinal epithelium beyond chloride absorption. Consistent with prior research, our study observed a marked reduction in DRA levels in TNF-α-stimulated Caco-2 cells. Nonetheless, HD treatment notably increased DRA expression and that of representative TJs and AJs, showcasing HD’s significant protective effect against TNF-α-induced damage to the Caco-2 cell monolayer barrier. However, silencing DRA with siDAR negated HD’s protective effect on intestinal permeability and its enhancement of TJs and AJs protein expression, without altering HNF-1β expression levels. This indicates DRA’s potential role downstream of HNF-1β. Ultimately, HD ameliorates intestinal barrier defects by upregulating TJs and AJs proteins, partly through activating DRA.

HNF-1β, a transcription factor belonging to the hepatocyte nuclear factor family, is primarily expressed in the liver and plays a crucial role in regulating systemic lipid, carbohydrate, and protein metabolism (Wang et al., 2024). While earlier research on HNF-1β concentrated on its impact on organ development (Yuan et al., 2009), recent investigations have proposed its significant function in UC. Our team previously observed a reduction in HNF-1β expression in clinical tissue samples from colorectal cancer patients (Y. Wang et al., 2020). Genomic analysis and visualization platforms have established that HNF-1β expression correlates positively with the overall survival rates of colorectal cancer patients. Moreover, HNF-1β contributes to the regulation of E-Cadherin and β-Catenin, pivotal in cell adhesion and intercellular junctions (Ferrè and Igarashi, 2019). Recent studies have also highlighted HNF-1β′s role in maintaining the integrity of renal cell structure and tight junctions by directly or indirectly influencing the components of the cell polarity complex (Tholen et al., 2023). These findings collectively suggest HNF-1β′s potential protective influence on intestinal barrier integrity. In this study, we have demonstrated that HD activates HNF-1β, which in turn reduces permeability and enhances the levels of AJs and TJs in models of DSS-induced experimental colitis in mice and TNF-α-induced monolayer barrier injury in Caco-2 cells. Departing from previous research, our study unveils a novel function of HNF-1β in mitigating intestinal inflammation by modulating intestinal epithelial permeability and mucosal immunity.

Numerous studies have elucidated the regulatory role of HNF-1β on DRA expression. For instance, the combined action of HNF-1β and HNF-1α has been shown to enhance DRA expression, influencing epithelial cell polarity, adhesion, cell division, differentiation, and intestinal water absorption (Uekuri et al., 2013). It has been documented that all-trans retinoic acid elevates DRA expression in intestinal epithelial cells through HNF-1β, offering therapeutic benefits for diarrhea resulting from intestinal inflammation or infectious diseases (Priyamvada et al., 2015). Building on these insights, AAV serotype 2/9 was utilized to suppress HNF-1β expression in mice, aiming to assess the impact of targeted HNF-1β gene inhibition on DSS-induced UC mice *in vivo*. Following the specific suppression of HNF-1β, the majority of HD’s protective actions were nullified; colitis symptoms intensified, intestinal permeability surged, and the expression of TJs and AJs was reduced. Additionally, DRA protein levels declined consequent to HNF-1β inhibition. The outcomes from both AAV and Lentivirus studies concur that HNF-1β is pivotal in HD’s protective influence on the intestinal barrier, with HNF-1β-induced upregulation of DRA playing a key role in mitigating colitis-induced epithelial barrier deficiencies through HD treatment.

This finding implies that HD may serve as a potential therapeutic strategy to help prevent and treat intestinal inflammation in UC patients by enhancing intestinal barrier function, providing important clinical implications for the development of new therapeutic options. This study has several significant advantages. Using the rescue experiment, which examined the effect of HD on DSS-induced ulcerative colitis after silencing HNF-1β, we demonstrated that HD indeed ameliorated UC through HNF-1β signaling. This suggests that HNF-1β is highly likely to be a target for HD. Nonetheless, we acknowledge certain limitations in this study. There are many factors that affect intestinal mucosal barrier function, such as MUC2, MAPK/PI3K, AMPK and other signaling pathways. However, how HD affects these factors remains unclear.

## 5 Conclusion

**FIGURE 11:**
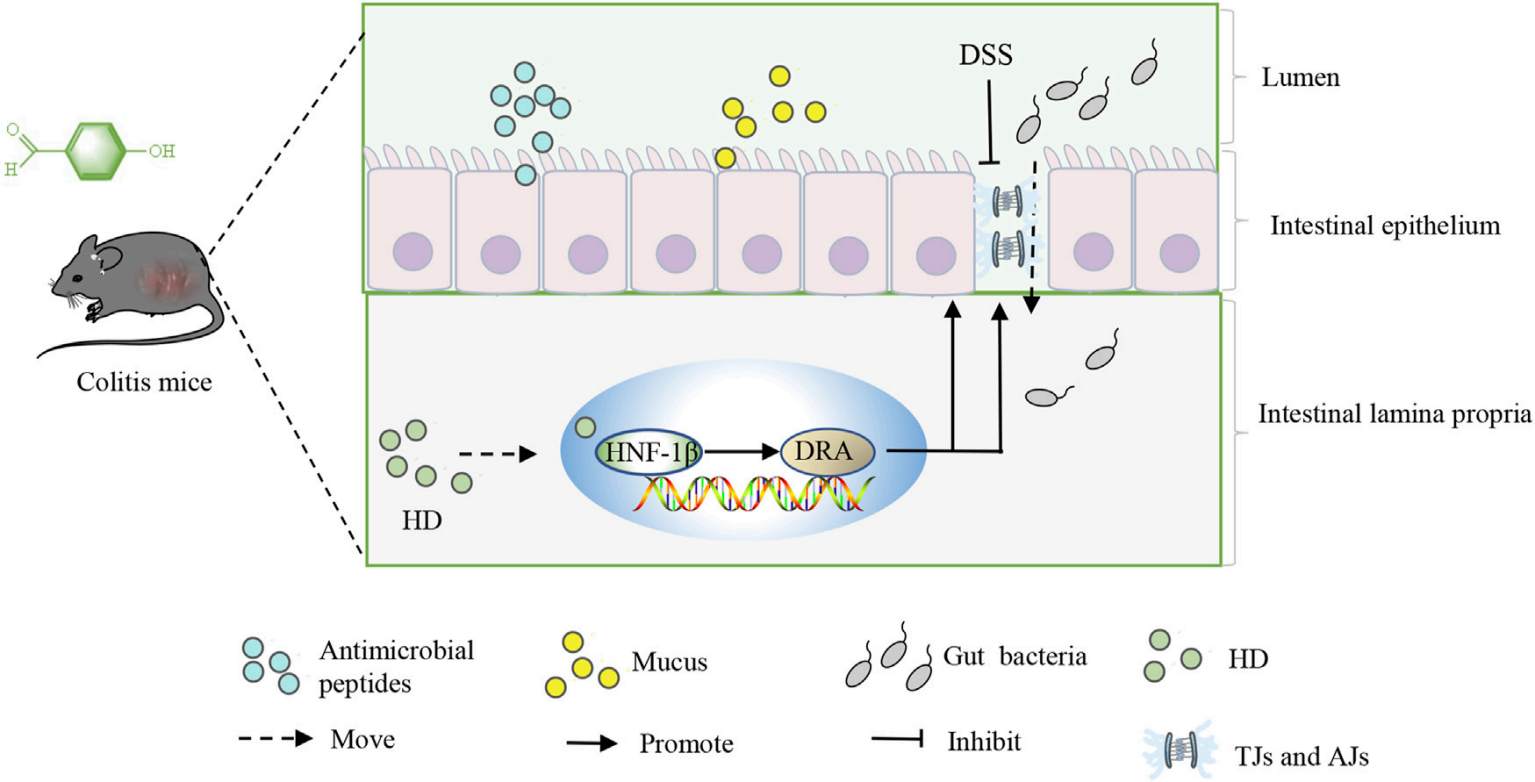
Outlines the mechanism by which HD acts, promoting the expression of TJs and AJs through activating the HNF-1β/SLC26A3 pathway, thus protecting the intestinal barrier from colitis-induced damages.

In summary, this study is the first to evaluate the pivotal role of HNF-1β in UC. Furthermore, our research indicates that HD enhances the expression of TJs and AJs through the activation of the HNF-1β/DRA pathway, effectively protecting the intestinal barrier from colitis-induced impairments (Figure 11). As HD may play a role in the treatment of UC through multiple mechanisms, our future works will explore the effect of HD on other signaling pathways in more detail.

## Data Availability

The original contributions presented in the study are included in the article/Supplementary Material, further inquiries can be directed to the corresponding authors.
