# Supplementary material for: p-hydroxy benzaldehyde attenuates intestinal epithelial barrier dysfunction caused by colitis via activating the HNF-1β/SLC26A3 pathway
Source: Front Pharmacol. 2024 Nov 22;15:1448863. doi: 10.3389/fphar.2024.1448863 (PMC11622497; doi:10.3389/fphar.2024.1448863)
Supplement: Supplementary file 1 [file Table1.pdf]

1 SUPPLEMENTARY MATERIALS AND METHODS

1.1 Evaluation of disease activity index (DAI)

The DAI was assessed daily, based on the mice's weight loss, stool consistency, and fecal hemorrhage, using the scoring criteria in Table S1. It was calculated as the average of these three parameters.

Table S1. Scoring system for calculating the disease activity index

Score	weight Loss (%)	stool consistency	fecal hemorrhage
0	none	normal	normal
1	1–5	loose stools (mild)	
2	5–10	loose stools (medium)	hemoccult positive
3	10–15	loose stools (high)	
4	>15	diarrhea	gross bleeding

1.2 Histological Analysis

Colonic segments measuring 0.5 cm were fixed in 4% paraformaldehyde for 24 h, embedded in paraffin, and then sectioned serially at 5 μm thickness using a microtome. The sections were stained with hematoxylin and eosin (H&E), dehydrated, dried, and mounted for H&E stained slides. Images were captured using a light field microscope (Mingmei, Guangzhou, China) and analyzed according to the scoring criteria in Table S2.

Table S2. Scoring system for histopathological changes in the colon

Score	Inflammation Severity	Inflammatory Locations	Crypt Damage
0	none	none	none
1	slight	mucosa	basal third damaged

2	moderate	mucosa and submucosa	basal two-thirds damaged
3	severe	transmural	only surface intact
4			entire crypt and epithelium lost

1.3 Myeloperoxidase (MPO) activity assay

MPO activity, indicative of neutrophil function, was measured in colonic tissues. The tissues, previously stored at −80°C, were homogenized in a ten-fold volume of cold PBS (0.1 M, pH 7.4) and centrifuged (3500 g, 15 min, 4°C) to obtain supernatants. MPO activity was then quantified using an MPO assay kit (Nanjing Jiancheng Bioengineering, Nanjing, China, A044-1-1).

1.4 Cell viability

Cell viability was determined by CCK-8 kit (Beyotime, Shanghai, China). Briefly, Caco-2 cells were placed in 96-well plates and incubated overnight at 37 ℃ with 5% CO_2 for 24 h. Then incubated with different concentrations of HD or TNF-α for 24 h. CCK8 reagent (10 %) was added to each well and incubated with cell at 37 ℃ for 1 h. The absorbance was read at 570 nm on a microplate reader (Epoch, BioTek, USA).

1.5 Detection of Cytokines

Colon tissue supernatants, previously collected as in the MPO activity assay, were used. Caco-2 cells co-treated with HD (1, 3, and 10 μM) and TNF-α (10 ng/mL) for 24 h yielded culture supernatants. The concentrations of cytokines such as IFN-γ and IL-13 in both colon tissue and cell culture supernatants were determined using ELISA kits, following the manufacturer's guidelines.

1.6 Immunohistochemistry (IHC)

The preparation and deparaffinization steps for the colon tissue section were the same as for H&E staining. The main steps were: endogenous peroxidase blocking agent was added to the tissue, incubated at room temperature for 10 min, and washed with double distilled water. Then, the slices were put into an EDTA antigen recovery solution, heated in a microwave oven on high heat for 8 minutes, naturally cooled for 8 minutes, heated on high heat for 8 minutes, and cooled to room temperature. Blocking solution with 5%BSA was added droppers and incubated at 37℃ for 30 min. Throw away excess liquid; do not wash. Appropriate dilutions of the primary antibody were added dropfold at 4℃ overnight. After removal, the cells were rewarmed at 37℃ for 30 min and washed 3 times with PBS (pH 7.2-7.6) for 5 min each time. Polymerized HRP anti-rabbit IgG was added dropfold and incubated at 37℃ for 30 min. Then, they were washed 3 times with PBS for 5 minutes each time. The DAB color development solution was dropped and observed under a microscope to control the reaction time. Wash with water. Mayor hematoxylin was added and incubated for 1 min at room temperature. Then, they were washed with PBS. The alkaline solution returned to blue. The slices were sealed with neutral gum, and the results were observed and photographed under the microscope.

1.7 Molecular docking analysis

Molecular docking to predict the binding affinity of HD to HNF-1β was conducted using CB-Dock (http://cao.labshare.cn/cb-dock/), a tool based on AutoDock Vina. This tool automatically identifies the ligand-receptor binding site, analyzing its central position and site. Protein and ligand files were obtained from the PDB (https:// www. rcsb. org/) and PubChem (https:// pubch em. ncbi. nlm. nih. gov/), respectively.

1.8 Molecular dynamics simulation analysis

Gromacs (version 2022.3) software wasemployed for molecular dynamics (MD) simulations. In the preprocessing of small molecules, AmberTools22 added the GAFF force field, while Gaussian 16W was used for hydrogenation and RESP potential calculation. The obtained potential data were integrated into the topology file of the MD system. The simulations were conducted at a constant temperature of 300K and atmospheric pressure (1 Bar). The Amber99sb-ildn force field, Tip3p water model as the solvent, and an appropriate number of Na+ ions were used to neutralize the total charge of the system. The system underwent energy minimization using the steepest descent method, followed by isothermal-isochoric (NVT) and isothermal-isobaric (NPT) equilibrations for 100,000 steps each, with a coupling constant of 0.1 ps and a duration of 100 ps. Subsequently, a free MD simulation was carried out for 5,000,000 steps with a step size of 2 fs, totaling 100 ns. The root mean square deviation (RMSD), root mean square fluctuation (RMSF), radius of gyration (Rg), and hydrogen bonds (H-bonds) from the MD simulation results were visualized using GraphPad Prism Version 9.0 (GraphPad Software, San Diego, CA, USA). The Gibbs free energy was calculated based on the RMSD and Rg values using a “bash” script, and 3D Gibbs free energy landscapes were plotted using Origin software (2021, OriginLab, USA).
